# Combined Effect of Exogenous Manganese Dioxide on the Remediation of Nonylphenol Pollution by *Arundo donax* Var. *versicolor*

**DOI:** 10.3390/toxics14100895

**Published:** 2026-10-08

**Authors:** Yongjie Huang, Xiaoyu Liang, Yuxi Jing, Xu Han, Yuting Cai, Jie Zhang

**Affiliations:** 1 School of Ecology and Environment, Anhui Normal University, Wuhu 241000, China; xiaoyuliang2001@163.com (X.L.); jingyuxi2000@163.com (Y.J.); 19920030923@163.com (X.H.); cnlblbq@163.com (Y.C.); zhjie1211@sina.com (J.Z.); 2 Collaborative Innovation Center of Recovery and Reconstruction of Degraded Ecosystem in Wanjiang Basin Co-Founded by Anhui Province and Ministry of Education, Anhui Normal University, Wuhu 241000, China; 3 Provincial Key Laboratory of Biotic Environment and Ecological Safety in Anhui, Wuhu 241000, China

**Keywords:** MnO_2_, nonylphenol (NP), *Arundo donax* var. *versicolor*, soil activity, soil microorganisms

## Abstract

Manganese dioxide (MnO_2_) is a potent oxidizing agent for degrading organic pollutants. To investigate the efficiency of MnO_2_ combined with *Arundo donax* var. *versicolor* (*A. donax* var. *versicolor*) in remediating nonylphenol-contaminated soil, pot experiments were conducted. Soil contaminated with nonylphenol (NP) (50 mg kg^−1^) was supplemented with varying MnO_2_ concentrations (0, 5, 10, 25, 50, 100, and 250 mg kg^−1^) and planted with *A. donax* var. *versicolor* seedlings. After 30 days of treatment, fundamental soil physicochemical properties, enzyme activities, microbial community composition and structure, and residual nonylphenol (NP) content were assessed. Results revealed that nonylphenol (NP) (50 mg kg^−1^) suppressed soil respiration while enhancing urease, sucrase, and phosphatase activities. MnO_2_ addition at concentrations ≤25 mg kg^−1^ elevated soil respiration and urease activity. Soil microbial community structure analysis across CK, N50, and M10 treatments demonstrated significant β-diversity alterations in N50 and M10 compared with CK. Firmicutes and Proteobacteria were the dominant phyla, while genera such as *Clostridium_sensu_stricto_12*, *Desulfitobacterium*, and *Citrifermentans* showed increased relative abundance under N50 and M10, but their functional role in nonylphenol (NP) degradation was not verified. MnO_2_ supplementation accelerated the reduction in extractable nonylphenol (NP) compared to the CK group, exhibiting a concentration-dependent increase. Therefore, optimal MnO_2_ application enhanced the removal of extractable nonylphenol (NP) in the combined *A. donax* var. *versicolor*–MnO_2_ system.

## 1. Introduction

Environmental Endocrine Disrupting Chemicals (EDCs) can disrupt endocrine signalling by interfering with hormone synthesis, receptor–ligand binding, or downstream signalling pathways [1]. Nonylphenol (NP), a typical endocrine disruptor, serves as a nonionic surfactant and plasticizer in food packaging materials and diverse industrial, agricultural, and domestic applications [2]. Nonylphenol (NP) pervades environmental matrices, including paddy soils, water, sediments, etc. [3]. Primary environmental nonylphenol (NP) sources stem from sewage treatment plant discharges, with prevalent detection in lakes, rivers, and sediments. Freshwater concentrations range from 0.7 ng L^−1^ to 634 μg L^−1^ [4]. Maximum nonylphenol (NP) concentrations in China’s Dongjiang River tributaries and mainstem reached 14.5 μg L^−1^ [5]. Against the U.S. Environmental Protection Agency (U.S. EPA) freshwater nonylphenol (NP) limit of 6.6 μg L^−1^, China’s surface water exhibits severe nonylphenol (NP) contamination, posing substantial environmental risks [6].

Nonylphenol (NP) constitutes a water-insoluble, persistent organic pollutant characterized by its stable chemical structure, strong lipophilicity, and non-degradability. These properties facilitate significant bioaccumulation and trophic transfer, amplifying their hazards and compromising the physiological integrity of animals, plants, microorganisms, and humans [7,8]. Nonylphenol (NP) exhibits multifaceted toxicity across diverse organisms. In mammals, it disrupts endocrine homeostasis, induces genotoxic effects, and impairs reproductive function. In plants, nonylphenol (NP) diminishes photosynthetic efficiency, regulates enzymatic activities, and alters stress-responsive gene expression patterns. Both acute high-dose and chronic low-dose exposures can lead to irreversible damage [9]. Begum et al. have shown that nonylphenol (NP) significantly reduces serum nitric oxide levels and suppresses sexual activity in mice [10]. Samanta et al. investigated NP’s toxicological mechanisms in zebrafish (Danio rerio), revealing that nonylphenol (NP) induced reactive oxygen species (ROS) accumulation, activated the mitogen-activated protein kinases (MAPK) pathway, triggered inflammation, and promoted ovarian follicular atresia, with higher accumulation in the ovarian tissue compared to muscle [11]. Maternal nonylphenol (NP) exposure resulted in delayed embryogenesis, developmental aberrations, and reduced offspring hatchability. Ke et al. showed that nonylphenol (NP) concentrations exceeding 0.1 mg L^−1^ inhibited the physiological growth of *Hydrocharis dubia* (Bl.) Backer, reduced chlorophyll content, and disrupted the antioxidant systems [12]. Yuan et al. found that nonylphenol (NP) treatment in the Sequencing Batch Reactor (SBR) significantly reduced the numbers of *Thauera* spp. and nitrifying bacteria *Nitrosomonas* spp., while concurrently increasing microbial diversity [13].

Nonylphenol (NP), a common environmental endocrine disruptor, exhibits distinct degradation mechanisms in different environments, resulting in disparate degradation rates and byproducts that contribute to environmental contamination at trace concentrations [14]. Nonylphenol (NP) has been identified as one of the 27 priority Persistent Toxic Substances (PTS) by the United Nations Environment Programme (UNEP) [15]. Furthermore, China’s “14th Five-Year Plan” New Pollutants Control Action Programme designates NPs as “new pollutants,” prohibiting their application in pesticide additives, etc. [6]. Phytoremediation represents an economical, ecologically benign, and sustainable approach for NP removal. The perennial wetland plant (*A. donax* var. *versicolor*) possesses extensive root architecture and strong immobilization force, prefers warm and well-lit environments, exhibits moisture and cold tolerance, and displays significant oxygen transport capacity and pollutant purification efficacy [16,17]. Manganese oxide (MnO_2_), a potent naturally occurring oxidant, manifests as a black amorphous powder or rhombohedral crystal characterized by substantial specific surface area and strong oxidizing properties, which is widely used as an oxidizing agent and adsorbent in industrial production and laboratories [18,19]. Notably, MnO_2_ demonstrates exceptional efficacy in pollutant degradation due to its high affinity for pollutants and many active sites [20,21]. Despite extensive research on MnO_2_-mediated organic pollutant removal, limited investigation has explored its integration with biomaterials to increase contaminant removal efficiency. Therefore, this study uses *A. donax* var. *versicolor* and MnO_2_ as combined remediation materials for nonylphenol (NP) pollution.

## 2. Materials and Methods

### 2.1. Materials and Reagents

#### 2.1.1. Test Soil

The test soil was collected from the 0–30 cm layer of natural soil behind the Ochre Mountain Campus of Anhui Normal University. The rear hill is part of the Ochre Mountain and is a typical volcanic landform. Its mountain body is mainly composed of andesite, and the soil appears reddish-brown. The soil type is usually yellow-brown soil, with a relatively coarse texture. The rear hill has dense vegetation, and its vegetation communities are rich in layers, ranging from tall trees to shrubs and herbaceous plants, forming a complex community structure. Fresh soil was air-dried, and then meticulously cleared of stones, vegetative debris, and large animal matter before being sieved through a 2 mm sieve, and 3 kg of sieved soil was placed in each pot for plant cultivation. The basic physicochemical properties of the soil are shown in Table 1, with nonylphenol (NP) content remaining undetectable.

#### 2.1.2. Test Plants and Reagents

*A. donax* var. *versicolor* is a perennial wetland grass with erect culms, lanceolate leaves, and distinctive yellow-white longitudinal stripes on the leaves. It has a well-developed rhizome system and high biomass. It prefers warm, humid, and sunny environments, is tolerant to waterlogging and cold, and is commonly found in riverbanks, wetlands, and pond margins. *A. donax* var. *versicolor* seedlings were purchased from Chongqing Flower Aixiao Horticulture Co., Ltd. (Chongqing, China) and subjected to testing following 15 days of acclimatization. Select uniform seedlings with an initial height of approximately 15 cm, and plant five seedlings per pot. MnO_2_ was purchased from Nanjing Wanqing Chemical Glassware Instrument Co., Ltd. (AR, Xilong Chemical, Nanjing, China). The water-soluble fertilizer, purchased from China Stanley Agricultural Group Co., Ltd. (Linyi, Shandong, China), contained primary nutrients (N + P_2_O_5_ + K_2_O) ≥ 60% with an N:P:K ratio of 1:1:1, and micronutrients (Zn + Fe + Mn + B) at 0.2–3.0%.

#### 2.1.3. Nonylphenol-Contaminated Soil Preparation

Nonylphenol (NP) was dissolved in 95% ethanol to prepare a 1 g L^−1^ stock solution. This solution was uniformly incorporated into the potting soil and allowed to equilibrate for 3 days to ensure complete ethanol evaporation. The blank control group was supplemented with an equivalent volume of ethanol and left to stand for three days until the ethanol had completely evaporated.

### 2.2. Experimental Design

Using the prepared polluted soil, each experimental pot was filled with 3 kg of soil (<2 mm) and amended with varying MnO_2_ concentrations. The experiment comprised nine treatments, and each was replicated three times, as shown in Table 2. Five uniformly growing *A. donax* var. *versicolor* seedlings were transplanted into each pot. Soil moisture was maintained through shallow irrigation (1–2 cm depth), and water-soluble fertilizer was applied as a basal dressing according to manufacturer dilution instructions. Seedlings were cultivated in a climate-controlled incubator under the following conditions for 30 days: 28/26 °C day/night temperature, 40% relative humidity, 9600 lx light, and a 14 h/10 h day/night photoperiod. Plant and soil parameters were subsequently assessed.

### 2.3. Indicator Measurement

#### 2.3.1. Determination of Soil Physical and Chemical Indexes

Soil physicochemical indicators were detected according to the Agricultural Industry Standard and Forestry Industry Standard of the People’s Republic of China [22]. The pH value was determined by using the potentiometric method with a soil-to-water ratio of 2.5:1. Soil organic matter (SOM) content was measured by the potassium dichromate–sulfuric acid method (NY/T 1121.6-2006). Alkali-hydrolyzable nitrogen (AN) was determined by using the alkaline dissolution diffusion method (LY/T 1228-2015). Soil available potassium (AK) was analyzed by flame photometer (NY/T 889-2004), while soil available phosphorus (AP) was determined by using the molybdenum–antimony inverse colorimetric method (NY/T 1121.7-2014).

#### 2.3.2. Determination of Effective Manganese Content in Soil

For manganese analysis, 10.00 g of air-dried soil sieved through a 1 mm mesh was placed into a 100 mL plastic wide-mouth bottle, followed by the addition of 20.0 mL of DTPA extractant, and then shaken at 25 °C for 2 h and filtered. The manganese concentration in the filtrate, blank solution, and standard solution was determined by using the flame atomic absorption spectrophotometer.

#### 2.3.3. Determination of Soil Respiration and Soil Enzyme Activities

Soil respiration was measured by using the alkali absorption method [23] (GB/T32720—2016). Urease activity was analyzed by the colorimetric technique [24,25]. Acid phosphatase was determined according to the protocol described by Tabatabai [26], while soil saccharase activity was analyzed by the colorimetric method using 3,5-dinitrosalicylic acid [27].

#### 2.3.4. High-Throughput Sequencing of Soil Microorganisms

This study established high-throughput sequencing for soil microorganisms. Only CK, N50, and M10 were selected for high-throughput sequencing, with each treatment comprising six replicates. Following freeze-drying, soil samples were entrusted to Guangzhou Kidio Biotechnology Co., Ltd. (Guangzhou, China). for microbial high-throughput sequencing to analyze soil bacterial community structure. Genomic DNA was extracted from samples, and the V3–V4 region of the *16S rRNA* gene was amplified using barcoded specific primers (341F: CCTACGGGGNGGCWGCAG; 806R: GGACTACHVGGGGTATCTAAT). PCR amplification system: Each 50 μL PCR reaction mixture contained 5 μL of 10 × KOD buffer, 5 μL of 2 mmol L^−1^ dNTPs, 3 μL of 25 mmol L^−1^ MgSO_4_, 1.5 μL of each primer (10 mmol L^−1^), 1 μL of KOD polymerase, and 100 ng of template DNA, with reactions performed in triplicate. Post-amplification, PCR products were excised and purified, and then quantified using a Qubit 3.0 fluorometer (Thermo Fisher Scientific, San Diego, CA, USA). The fragmentation and length of the *16S rRNA* gene PCR products were detected by 1% agarose gel electrophoresis. Qualified amplicons underwent purification, quantification, and library construction before sequencing on the NovaSeq 6000 platform (Illumina, San Diego, CA, USA) following quality inspection. Sequencing results underwent pre-processing: raw reads were filtered using the Trimmomatic v0.33 software, followed by primer sequence identification and removal with cutadapt 1.9.1 software to obtain primer-free clean reads. Usearch v10 software performed overlap-based assembly of clean reads per sample, with sequences filtered by region-specific length parameters. Bacterial sequences were denoised and chimera-removed by using UCHIME algorithm, Unoise 3 algorithm in Usearch (version 11.0.667), and UPARSE algorithm in Usearch (version 11.0.667), respectively. Operational taxonomic units (OTUs) were clustered based on sequence composition to determine species classification, generating the final validated dataset.

#### 2.3.5. Determination of Extractable Nonylphenol (NP) Content in Soil

Soil nonylphenol (NP) content was determined by Beijing Qingxi Technology Research Institute. High-performance liquid chromatography–mass spectrometry (HPLC-MS) analysis was conducted under the following conditions: Thermo Fisher U3000 coupled with MSQ Plus; column: Xtimate UHPLC C18 (3 μm, 2.1 × 100 mm); mobile phases: A: acetonitrile; B: 0.05% aqueous ammonia; gradient elution: 0–1 min (10% A, 90% B), 3–7 min (90% A, 10% B), 7–10 min (10% A, 90% B); column temperature: 45 °C; flow rate: 0.3 mL min^−1^; injection volume: 10 μL. Mass spectrometry parameters: probe temperature: 350 °C; cone voltage: 75 V; needle voltage: 3.5 kV; negative ion mode; quantitative ion (M-H): *m/z* 219. Standard preparation: Accurately weigh 10.00 mg of reference standard (precision ± 0.00001 g), dissolve in methanol, and dilute to 10 mL in a volumetric flask. Preparation of working standards was performed by serial dilution to appropriate concentrations. Sample treatment: We weighed 2.0 g of soil sample into a 10 mL centrifuge tube, added 1 mL of deionized water and 1 mL of acetonitrile, and then sonicated the solution for 30 min at room temperature. We centrifuge at 1000 r min^−1^ for 5 min, and transferred the supernatant to a new 10 mL centrifuge tube containing 1 g sodium chloride. The supernatant was shaken vigorously for 1 min, and then allowed to undergo phase separation for 30 min at room temperature. The entire supernatant layer was collected and then concentrated to near dryness. We reconstituted the residue in 1 mL of acetonitrile, mixed thoroughly, and filtered through a 0.22 μm filter membrane prior to analysis. The high concentration of the samples exceeding the calibration range was diluted appropriately before analysis.

### 2.4. Data Analysis

Data are presented as mean ± standard deviation (n = 3). Statistical significance was determined through one-way ANOVA followed by Tukey’s HSD test (*p* < 0.05). Pearson correlation was used to assess relationships among variables. Statistical analyses were performed using SPSS 19.0 (IBM, Armonk, NY, USA) and Excel 2019 (Microsoft, Redmond, WA, USA); figures were generated using Origin 2022 (OriginLab, Northampton, MA, USA).

To identify indicator taxa associated with each treatment, linear discriminant analysis effect size (LEfSe) was performed using the online Omicsmart platform (https://www.omicsmart.com/home.html#/). Taxa with LDA > 3.5 and *p* < 0.05 were considered significantly enriched indicator taxa. LEfSe analysis was restricted to the CK, N50, and M10 treatments.

## 3. Results

### 3.1. Effect of Manganese Dioxide (MnO_2_) on the Physicochemical Properties of Inter-Root Soil of A. donax Var. versicolor Under Nonylphenol (NP) Stress

**Figure 1:**
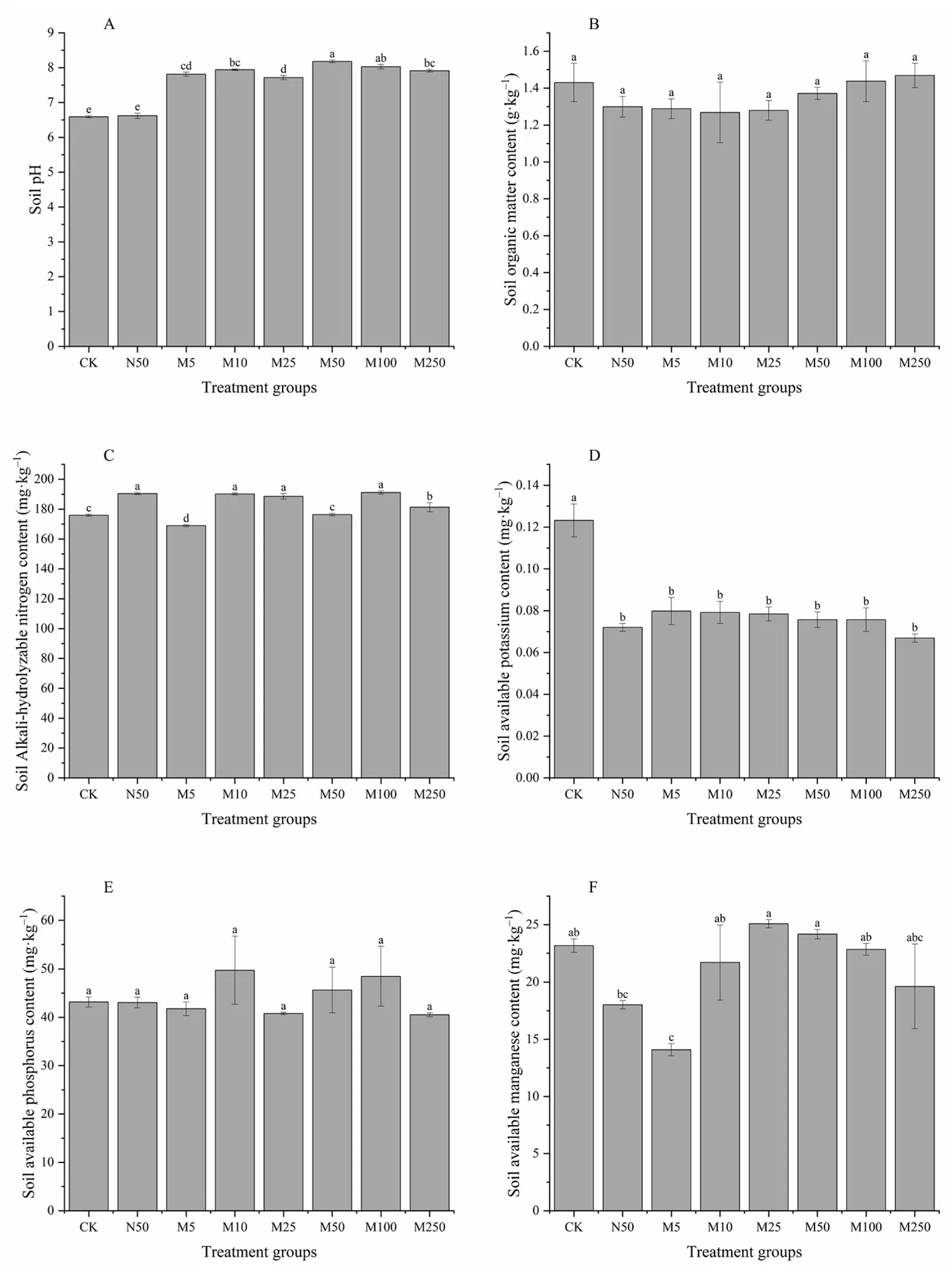
Effects of MnO_2_ on soil pH (**A**), organic matter (**B**), alkali-hydrolyzable nitrogen (**C**), available potassium (**D**), available phosphorus (**E**), and available manganese (**F**) under nonylphenol (NP) stress. Note: Different lowercase letters above the bars indicate significant differences among treatments at the *p* < 0.05 level.

Under identical nonylphenol (NP) stress concentration, varying MnO_2_ additions affected soil pH, organic matter, alkali-hydrolyzable nitrogen, and available potassium content (Figure 1). There was no significant difference in the soil pH value in N50 compared with that of CK, whereas MnO_2_ addition increased pH, with M50 exhibiting the highest value, which was 1.24 times higher than that of CK. Soil organic matter content showed no significant difference among treatments, following the hierarchy M250 > M100 > CK > M50 > N50 > M5 > M25 > M10. Alkali-hydrolyzable nitrogen (ADN) content was shown as M100 > N50 > M10 > M25 > M250 > M50 > CK > M5. M100 was 1.08 times more than that of CK, while M10 showed a 3.97% reduction compared with CK. Available potassium content was expressed as CK > M5 > M10 > M25 > M50 > M100 > N50 > M250, with M5, M10, M25, M50, M100, N50, and M250 showing respective decreases of 35.23%, 35.74%, 36.40%, 38.59%, 38.60%, 41.56%, and 45.73% compared to CK (*p* < 0.05). No significant differences in soil available phosphorus content among these treatments. Soil effective manganese content was lowest in M5, suggesting an initial increase followed by a decrease with rising MnO_2_ addition, specifically ordered as M25 > M50 > CK > M100 > M10 > M250 > N50.

### 3.2. Effect of Manganese Dioxide (MnO_2_) on Soil Enzyme Activities Under Nonylphenol (NP) Stress

**Figure 2:**
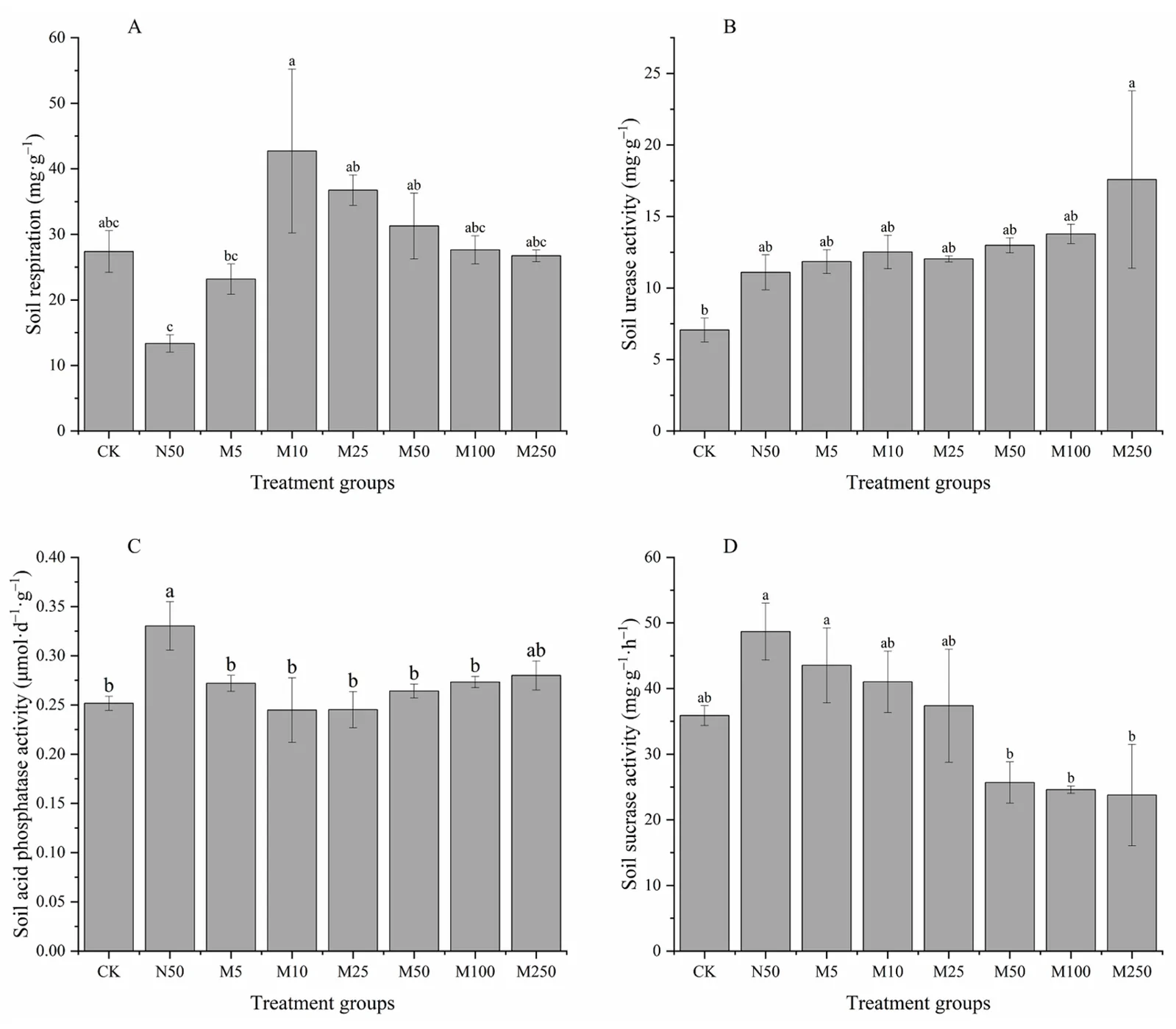
Effect of MnO_2_ on soil respiration (**A**), soil urease activity (**B**), soil acid phosphatase (**C**), and soil sucrase activity (**D**) under nonylphenol (NP) Stress. Note: Different lowercase letters above the bars indicate significant differences among treatments at the *p* < 0.05 level.

Under the same nonylphenol (NP) stress concentrations, varying MnO_2_ additions markedly affected soil respiration intensity, urease and sucrase activities, and acid phosphatase activity (Figure 2). Soil respiration intensity was lowest in N50, exhibiting a 51.21% decrease compared with that in CK. Respiration initially enhanced but subsequently weakened with increasing MnO_2_, peaking in M10 at 1.56 times the CK rate. Soil enzyme activities showed a rising trend with MnO_2_ addition, and M250 achieved maximal urease activity, significantly surpassing CK by 2.48 times (*p* < 0.05). Acid phosphatase activity was highest in N50, 1.31 times that of CK (*p* < 0.05), but exhibited a biphasic response to MnO_2_, decreasing to its lowest point in M10 by 2.67% before rising as compared to CK. Conversely, sucrase activity peaked at N50, 1.35 times that of CK, but progressively decreased with MnO_2_ addition, soil sucrase activity reaching its nadir in M250 by 33.74% as compared to CK.

### 3.3. Changes in Soil Bacterial Community Diversity Among Ck, N50, and M10 Treatments

The changes in α-diversity of the bacterial community among CK, N50, and M10 treatments are shown in Table 3. The treatments demonstrated no significant differences regarding the number of OTUs and Ace and Chao1 indices. Compared with CK, the N50 and M10 treatments showed increases in Shannon indices by 6.75% and 10.27%, respectively, while Simpson indices increased by 1.06% and 2.13%, respectively. These findings suggest that the addition of both nonylphenol (NP) and MnO_2_ altered the bacterial community abundance in the soil environment.

**Figure 3:**
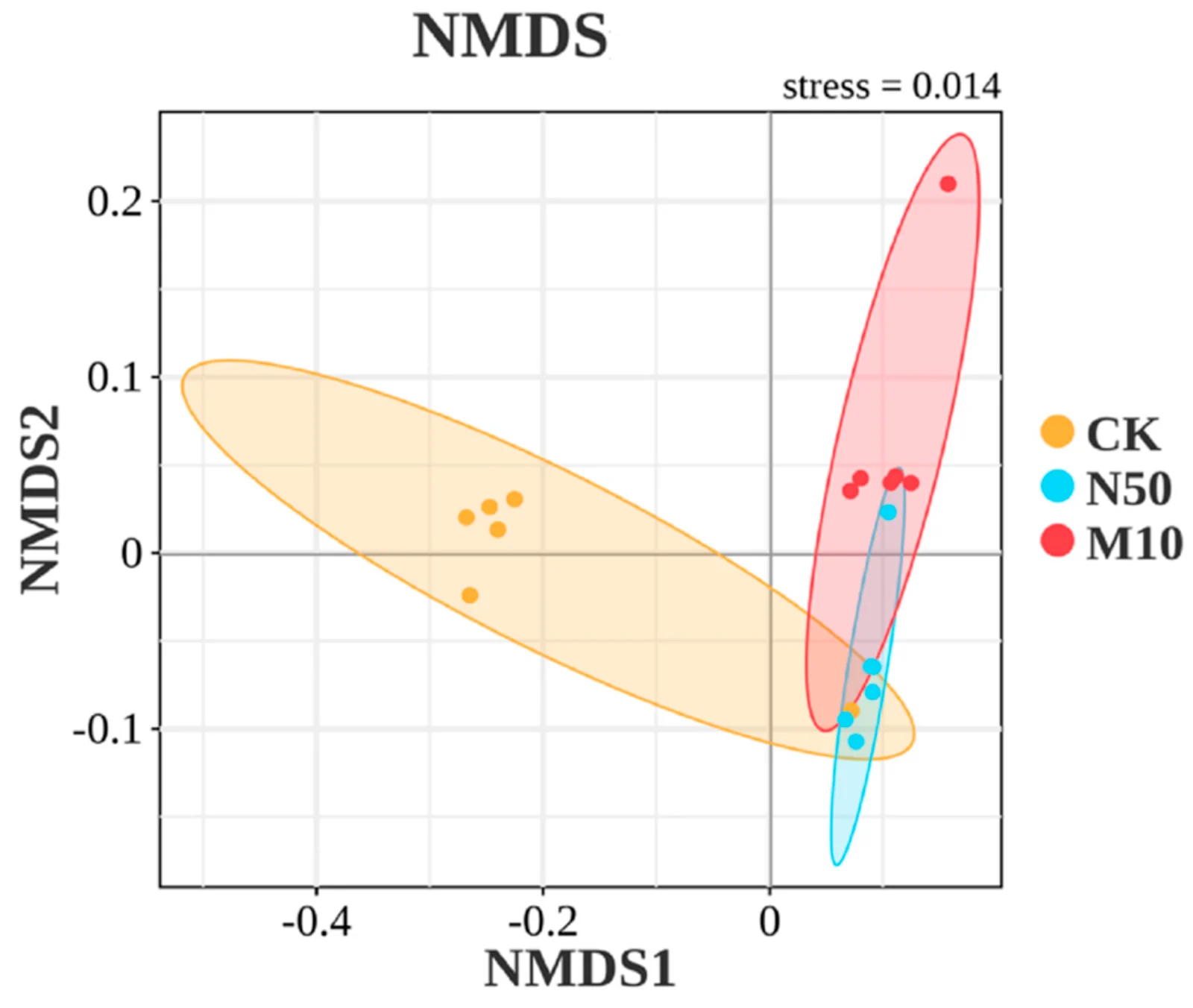
Effects of different treatments on β-diversity of soil bacterial communities.

The β-diversity of soil bacterial communities across CK, N50, and M10 treatments was analyzed by using Bray-based non-metric multidimensional scaling (NMDS) (Figure 3). The results showed distinct bacterial community structures in N50 and significantly divergent communities in M10 compared with CK (*p* < 0.05). Meanwhile, the microbial communities between N50 and M10 differed substantially (*p* < 0.05).

### 3.4. Changes in the Composition of Soil Bacterial Communities Among Ck, N50, and M10 Treatments

**Figure 4:**
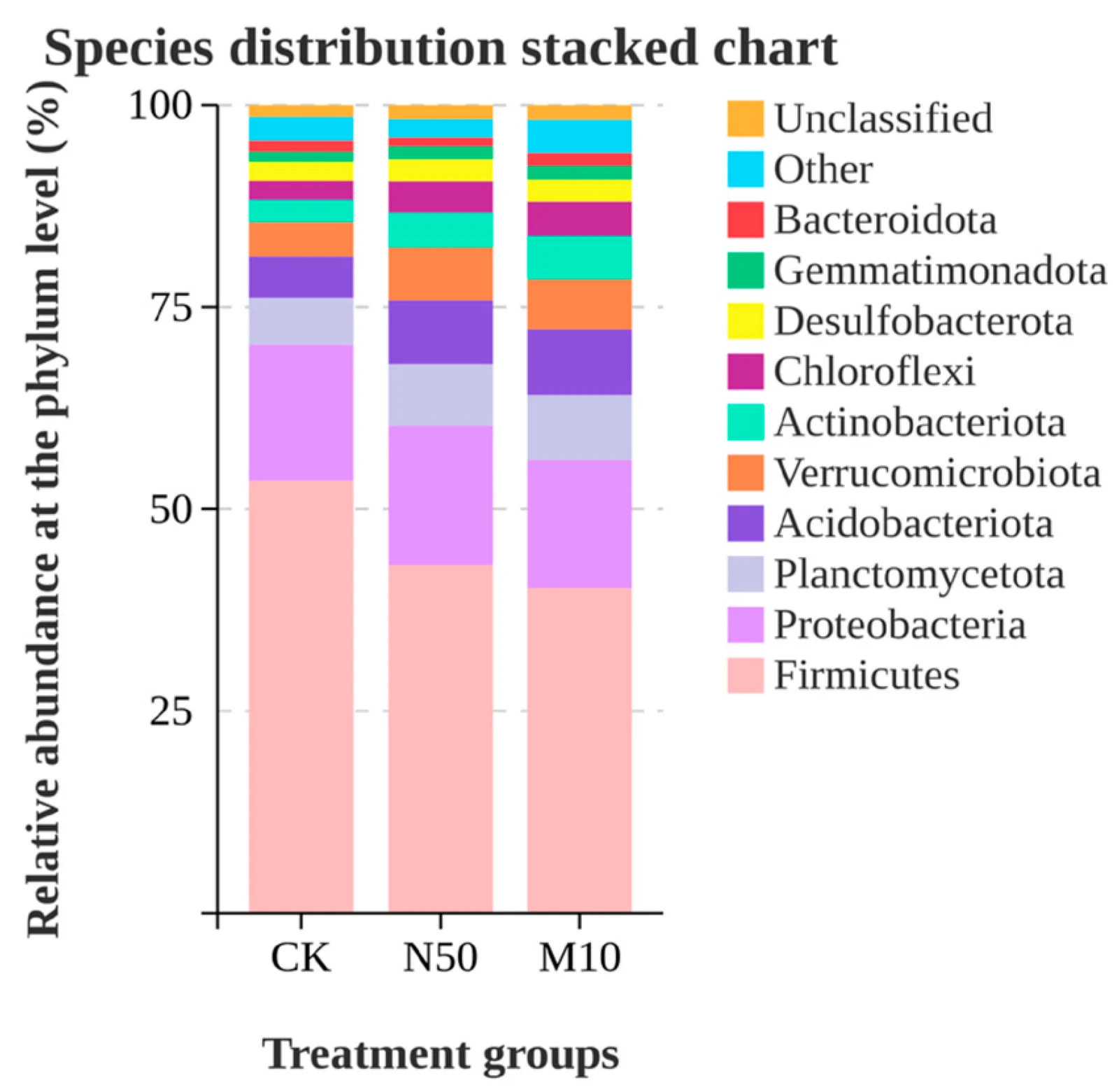
Effects of different treatments on soil bacterial community composition at the phylum level.

The soil bacterial community composition among CK, N50, and M10 treatments at the phylum level is shown in Figure 4. Within the CK, N50, and M10 groups, Firmicutes, Proteobacteria, Planctomycetota, and Acidobacteriota emerged as dominant phyla, whose relative abundances were over 5%, accounting for 81.24%, 75.79%, and 72.21% of the total sequences, respectively. Subsequently, abundant phyla included Verrucomicrobiota, Actinobacteriota, Chloroflexi, Desulfobacterota, Gemmatimonadota, and Bacteroidota. Specifically, Firmicutes occupied 53.44%, 43.01%, and 40.16% in CK, N50, and M10; Proteobacteria constituted 16.92%, 17.25%, and 15.89%; Planctomycetota represented 5.71%, 7.67%, and 8.07%; while Acidobacteriota made up 5.16%, 7.84%, and 8.08% across these groups. Nonylphenol (NP) treatment decreased the relative abundance of Firmicutes compared to CK but increased the relative abundance of Proteobacteria, Planctomycetota, and Acidobacteriota. MnO_2_ addition decreased the relative abundance of Firmicutes and Proteobacteria compared to CK, yet increased the relative abundance of Planctomycetota and Acidobacteriota.

**Figure 5:**
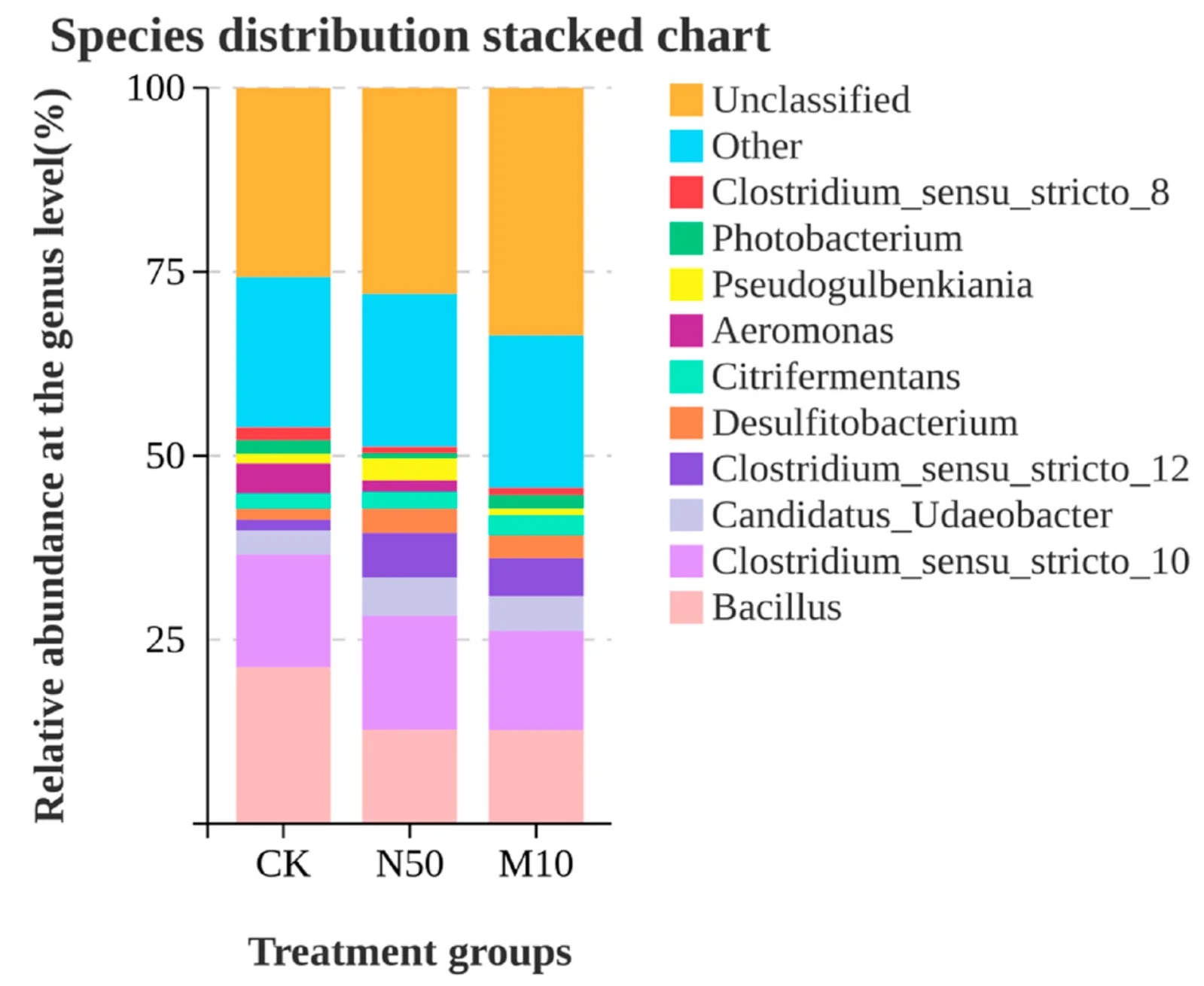
Effects of different treatments on soil bacterial community composition at the genus level.

The relative abundance of bacterial genera among CK, N50, and M10 treatments is shown in Figure 5. Excluding unclassified and other taxa, the ten most abundant genera across CK, N50, and M10 treatments were *Bacillus*, *Clostridium_sensu_stricto_10*, *Candidatus_Udaeobacter*, *Desulfitobacterium*, *Clostridium_sensu_stricto_12*, *Citrifermentans*, *Aeromonas*, *Pseudogulbenkiania*, *Photobacterium*, and *Clostridium_sensu_stricto_8*. The distribution patterns revealed that *Bacillus* constituted 21.31%, 12.75%, and 12.69% in CK, N50, and M10 samples, respectively. *Clostridium_sensu_stricto_10* comprised 15.27%, 15.51%, and 13.52%; *Candidatus_Udaeobacter* accounted for 3.25%, 5.17%, and 4.72%; *Clostridium_sensu_stricto_12* represented 1.49%, 6.07%, and 5.14%; and *Desulfitobacterium* constituted 1.43%, 3.29%, and 3.13%. Additionally, *Citrifermentans* represented 2.09%, 2.34%, and 2.65%; *Aeromonas* 4.08%, 1.48%, and 0.02%; *Pseudogulbenkiania* 1.30%, 3.04%, and 0.91%; *Photobacterium* 1.85%, 0.65%, and 1.86%; and *Clostridium_sensu_stricto_8* 1.72%, 0.88%, and 0.99%. Compared with CK, the N50 group reduced the relative abundance of *Bacillus*, *Aeromonas*, Photobacterium, and *Clostridium_sensu_stricto_8*, while showing the increased relative abundance of *Clostridium_sensu_stricto_10*, *Candidatus_Udaeobacter*, *Clostridium_sensu_stricto_12*, *Desulfitobacterium*, *Citrifermentans*, and *Pseudogulbenkiania*. Similarly, the M10 treatment demonstrated decreased relative abundance of *Bacillus*, *Clostridium_sensu_stricto_10*, *Aeromonas*, *Pseudogulbenkiania*, *and Clostridium_sensu_stricto_8*, with concurrent increases in *Candidatus_Udaeobacter*, *Clostridium_sensu_stricto_12*, *Desulfitobacterium*, *Citrifermentans*, and *Photobacterium*.

**Figure 6:**
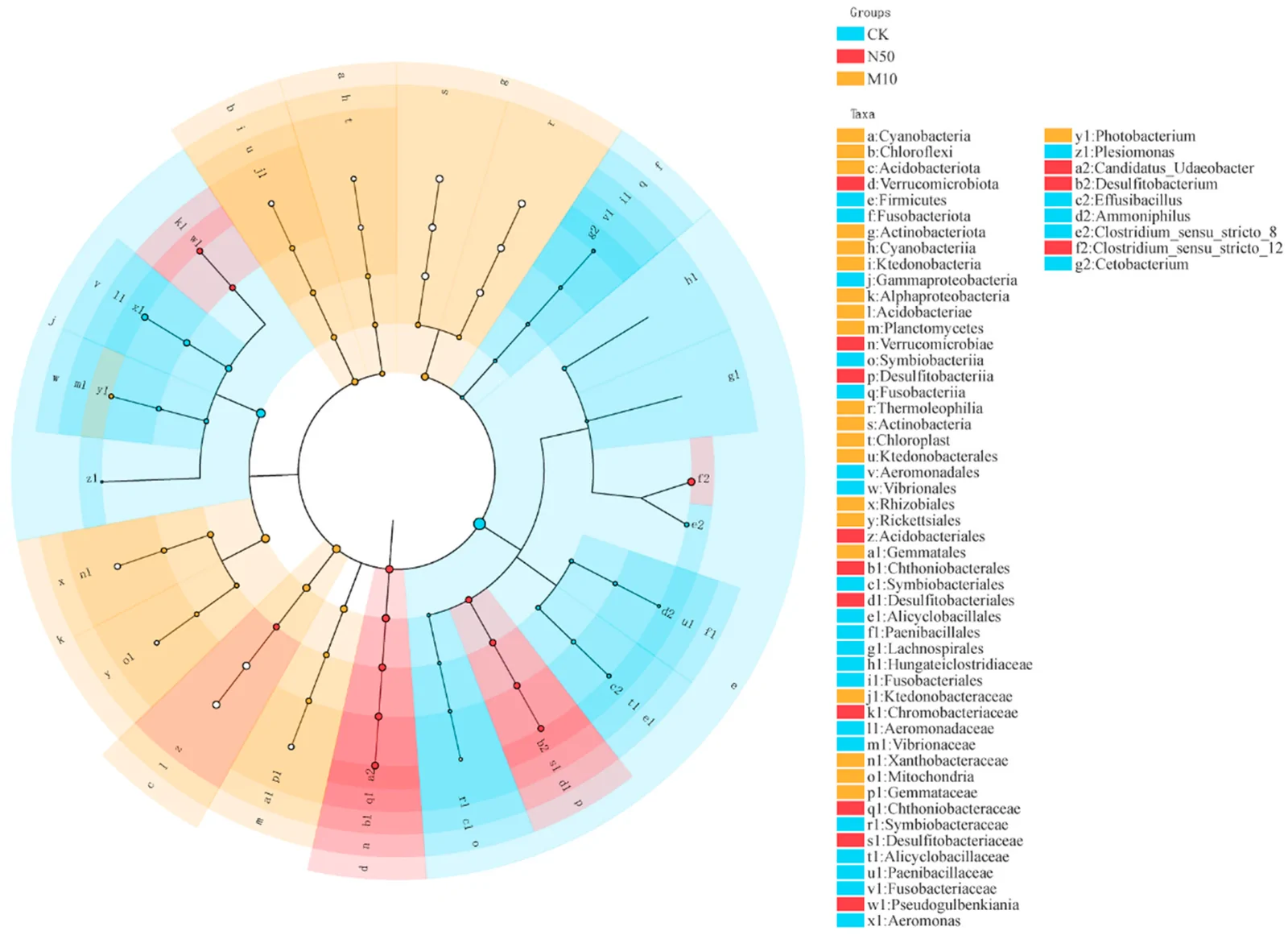
LEfSe analysis (LDA > 3.5) of bacterial community composition under different treatments in the soil.

LEfSe analysis was further employed to identify indicator taxa among CK, N50, and M10 treatments (Figure 6). At the phylum level, Cyanobacteria, Chloroflexi, Acidobacteriota, and Verrucomicrobiota were significantly enriched in M10, whereas Firmicutes and Actinobacteriota were enriched in CK. N50 was characterized by the enrichment of Acidobacteriae and Planctomycetes. At the genus level, the M10 treatment was characterized by the significant enrichment of *Candidatus_Udaeobacter*, *Desulfitobacterium*, *Clostridium_sensu_stricto_12*, and *Cetobacterium* (LDA > 3.5, *p* < 0.05). In contrast, *Aeromonas* and *Photobacterium* were enriched in CK.

### 3.5. Association Analysis Between Changes in Soil Microbial Community Structure and Environmental Factors Among Ck, N50, and M10 Treatments

**Figure 7:**
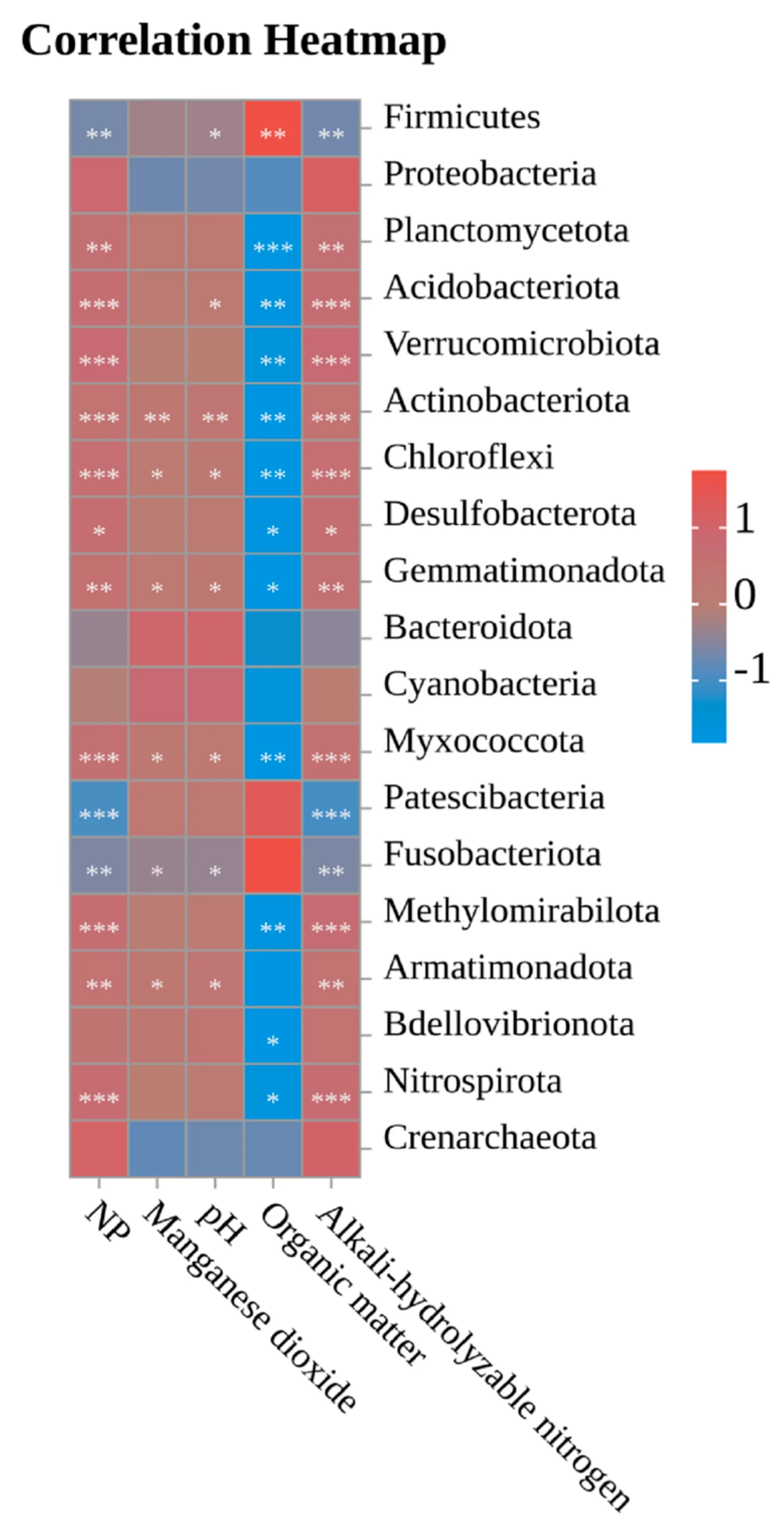
Association analysis between soil bacterial community and environmental factors (phylum level). Note: * represents *p* < 0.05; ** represents *p* < 0.01; *** represents *p* < 0.001.

Figure 7 illustrates the association analysis between soil bacterial community structure changes and environmental factors among CK, N50, and M10 treatments at the genus level. The results showed that Firmicutes exhibited a significant negative correlation with nonylphenol (NP) content and alkali-hydrolyzable nitrogen, while demonstrating a significant positive correlation with the organic matter content. Conversely, Acidobacteria, Planctomycetota, Chloroflexi, Verrucomicrobiota, and Actinobacteria displayed significant positive correlations with nonylphenol (NP) and alkali-hydrolyzable nitrogen content, but showed significant negative correlations with organic matter content.

**Figure 8:**
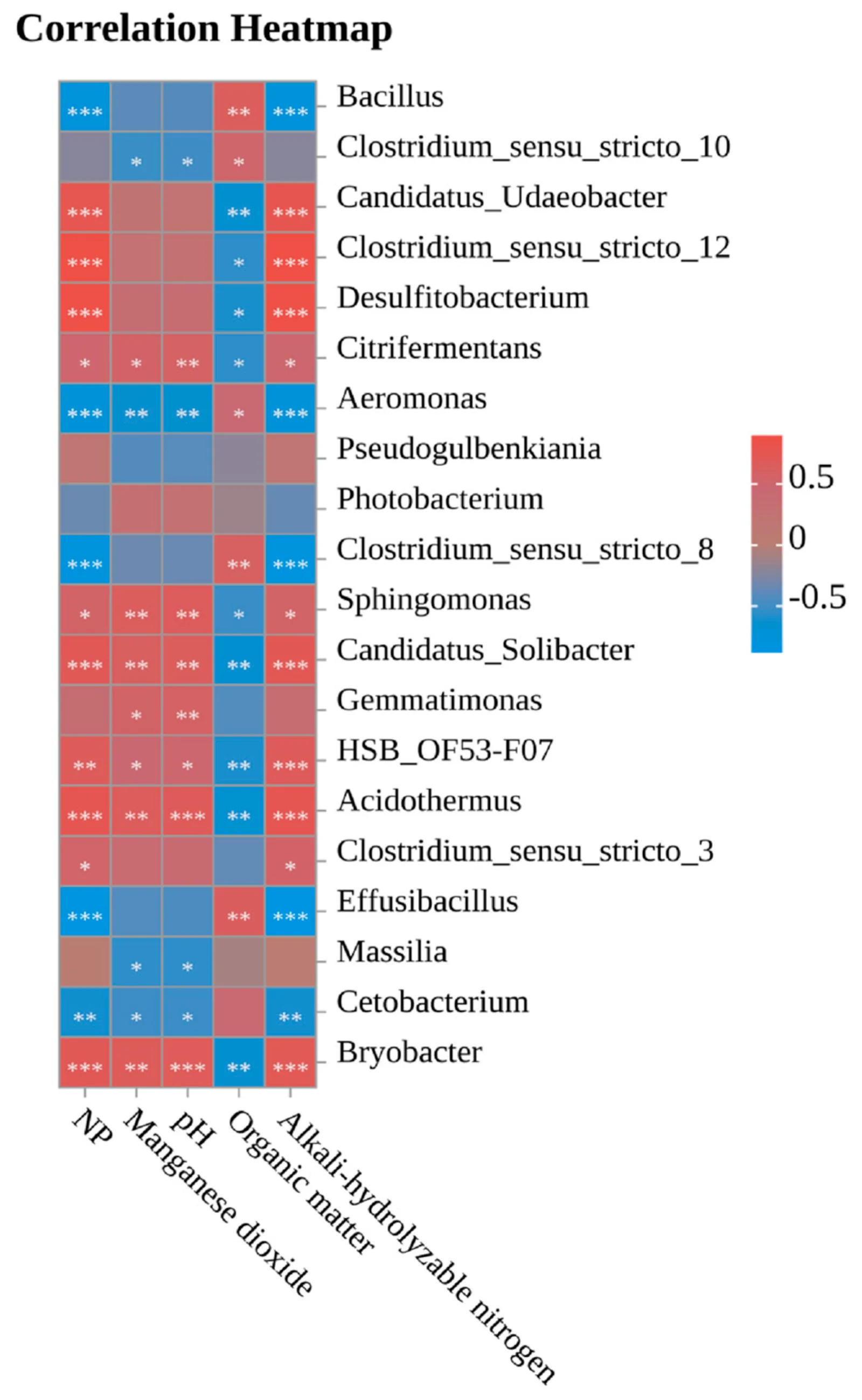
Association analysis between soil bacterial community and environmental factors (genus level). Note: * represents *p* < 0.05; ** represents *p* < 0.01; *** represents *p* < 0.001.

The association between soil bacterial community structure and environmental factors among CK, N50, and M10 treatments at the genus level is shown in Figure 8. *Bacillus* and *Clostridium_sensu_stricto_8* showed significant negative correlations with nonylphenol (NP) and alkali-hydrolyzable nitrogen content, while exhibiting significant positive correlations with organic matter content. In contrast, *Candidatus_Udaeobacter* and *Clostridium_sensu_stricto_12* showed significant positive correlations with nonylphenol (NP) and alkali-hydrolyzable nitrogen content, and significant negative correlations with organic matter content.

These correlations are associations and do not establish causality. The observed relationships may be confounded by pH, organic matter, available nitrogen, plant growth, MnO_2_-induced redox changes, and other treatment effects.

### 3.6. Nonylphenol (NP) Residue Content in Soil

**Figure 9:**
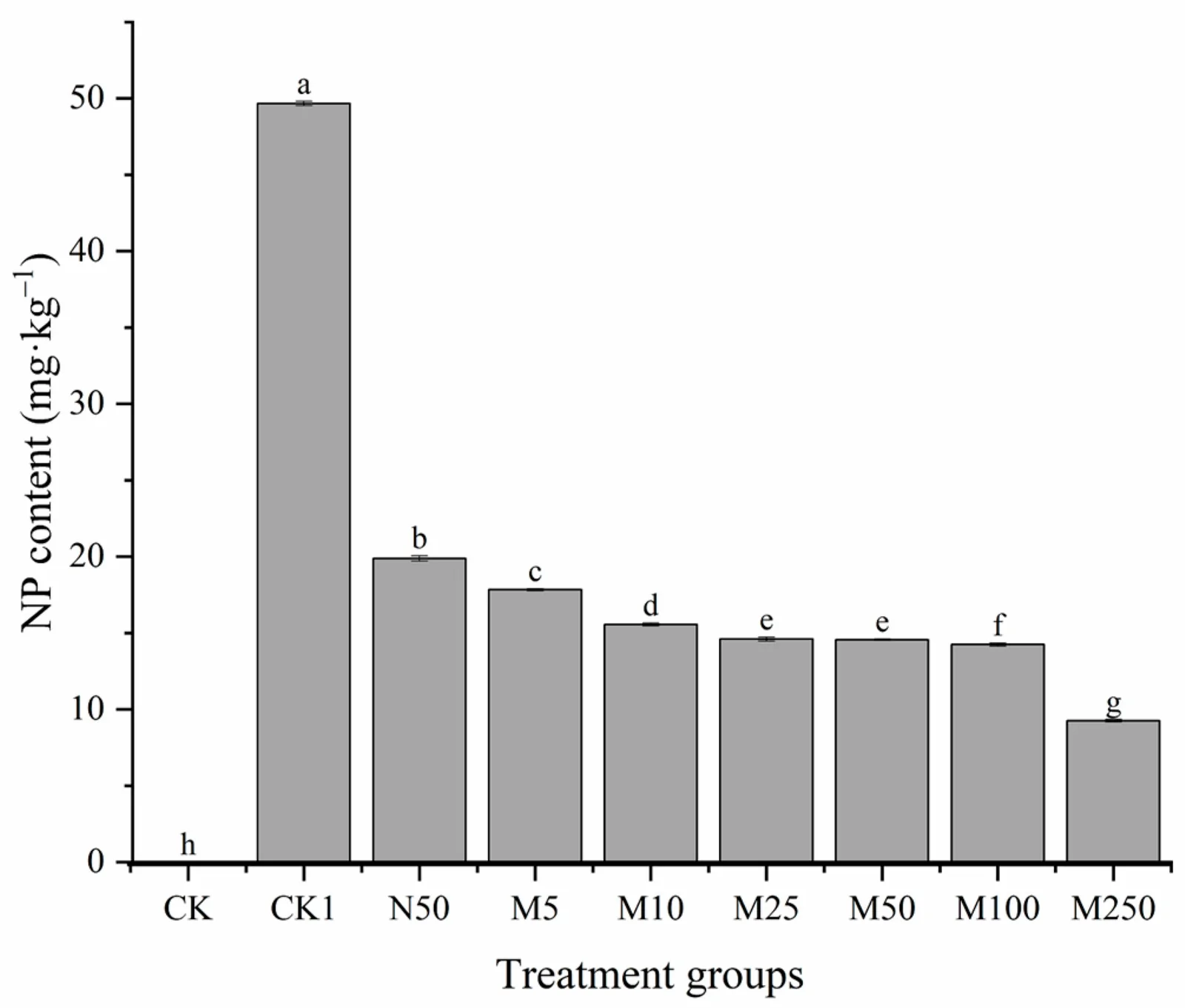
Residual nonylphenol (NP) content. Note: Nonylphenol (NP) content refers to extractable nonylphenol (NP) recovered by the HPLC-MS procedure and does not represent total or mineralized nonylphenol (NP). Note: Different lowercase letters above the bars indicate significant differences among treatments at the *p* < 0.05 level.

Figure 9 illustrates the residual extractable nonylphenol (NP) content in soil. The CK1 group exhibited the highest content at 49.67 mg kg^−1^. The cultivation of *A. donax* var. *versicolor* combined with MnO_2_ supplementation substantially reduced the extractable nonylphenol (NP) content, which ultimately reached 28.48% of the initial value. With the exception of CK, the treatments demonstrated the following removal sequence—N50 > M5 > M10 > M25 > M50 > M100 > M250—corresponding to reduction rates of 59.97%, 64.08%, 70.60%, 70.68%, 71.33%, and 81.35%, respectively, compared with CK1. These findings indicated that MnO_2_ addition enhanced the removal of extractable nonylphenol (NP) in the combined *A. donax* var. *versicolor*–MnO_2_ system.

## 4. Discussion

### 4.1. Soil Chemical and Enzyme Responses

Soil physical and chemical properties are important indicators of soil quality, with soil pH standing as a fundamental measure of soil health that significantly affects soil enzyme activity, microbial community composition, and nutrient cycling [28,29]. Monitoring these health indicators is essential for maximizing crop yields, implementing soil conservation practices, and regulating climate change [30]. This study showed that nonylphenol (NP) addition minimally influenced soil pH, whereas MnO_2_ addition observably increased it. It is possible that plants enhance alkaline root exudation to adapt to exogenous Mn toxicity, leading to higher soil pH values [31]. No significant difference emerged in soil organic matter content among treatments, probably due to MnO_2_ participation in organic matter decomposition processes. Given that MnO_2_ could not directly participate in the soil phosphorus cycle, the soil available phosphorus content showed no significant difference under the regulation of the rhizosphere microorganisms of *A. donax* var. *Versicolor*. And soil available potassium content of the soil in each treatment group was lower than that of CK. This consistent decline may result from increased potassium uptake by *A. donax* var. *versicolor* under nonylphenol (NP) stress, MnO_2_-induced changes in soil pH and surface chemistry, microbial potassium immobilization, or altered root exudation.

Soil enzymes constitute vital components of soil ecosystems, with fluctuations in their activities serving as critical indicators for evaluating soil fertility, monitoring pollution remediation, and forecasting alterations in soil quality [32]. These enzymes additionally modulate the interplay between soil nutrients and microorganisms [33]. Soil respiration represents an integrated process wherein plant roots, soil fauna, and microorganisms metabolize organic matter, generating carbon dioxide; it functions as a significant indicator of aggregate soil microbial activity [34]. The present investigation revealed that nonylphenol (NP) diminished soil respiration intensity—a finding inconsistent with Zaytseva et al.’s research [35]. This discrepancy potentially arises from nonylphenol (NP) concentrations inhibiting soil microbial activity, thereby counteracting the respiratory compensation induced by supplementary carbon inputs and ultimately suppressing respiration intensity. Soil urease catalyzes urea hydrolysis into ammonium nitrogen, which plants and soil organisms can assimilate; its activity signifies the soil’s capacity to transform organic nitrogen into bioavailable forms and sustain inorganic nitrogen pools [36]. In this study, significant urease activity differences emerged solely between hydrolyzable CK treatments. Combined with alkali-hydrolyzable nitrogen measurements, these results indicate MnO_2_ exerts minimal influence on the soil nitrogen cycle. Kuzikova et al. demonstrated that nonylphenol (NP) presence reduces soil microbial mineralization intensity, impeding nitrogen fixation—a conclusion contrasting with our findings, possibly attributable to the lower nonylphenol (NP) dosage (300 mg kg^−1^) employed by Kuzikova, which consequently elicited diminished microbial toxicity [37]. Soil acid phosphatase (ACP) plays a pivotal role in governing organic phosphorus mineralization and biogeochemical cycling within terrestrial ecosystems. ACP activity directly determines soil phosphorus availability, thereby enhancing plant productivity and preserving community diversity [38]. This study observed elevated ACP activity following nonylphenol (NP) addition, likely due to microbial stimulation, whereas acid phosphatase showed a biphasic response to MnO_2_, with the highest activity in N50 and the lowest in M10. Soil sucrase, which catalyzes sucrose hydrolysis into glucose and fructose, provides essential energy for microorganisms, plants, and animals; its activity serves as a key carbon cycle indicator [39]. Here, sucrase activity increased with nonylphenol (NP) addition but decreased with MnO_2_ treatment. These divergent patterns indicate that MnO_2_ does not uniformly stimulate soil enzyme activity and may differentially affect carbon and phosphorus cycling. Therefore, the enzyme data should be interpreted as dose-dependent and enzyme-specific responses rather than as general evidence of improved soil functioning.

### 4.2. Microbial Community Changes

Soil bacteria constitute integral components of soil ecosystems, with bacterial communities being indispensable for sustaining essential ecosystem functions, including supporting aboveground biomass production and facilitating nutrient cycling [40]. In this investigation, nonylphenol (NP) contamination and MnO_2_ addition (N50 and M10) exhibited negligible effects on bacterial OTU numbers, Chao1 index, and Ace index. Although Shannon and Simpson indices demonstrated marginal increases compared to the control group (CK), soil microbial α-diversity remained statistically indistinguishable from CK, while β-diversity diverged significantly. These findings indicate that while MnO_2_ and nonylphenol (NP) supplementation did not alter microbial species richness, evenness, or overall diversity, they substantially modified community composition, thereby exerting a pronounced influence on bacterial community differentiation. One possible reason is that oxidizing agents produced strong oxidative free radicals, which could break the cell structure of some microorganisms in soil and kill some microorganisms, causing the difference in microbial community structure [41]. This observation also aligns with Chen’s research demonstrating NP’s minimal impact on soil α-diversity yet substantial effect on soil β-diversity [42]. The introduction of nonylphenol (NP) likely promoted the proliferation of microbial species capable of degrading or tolerating the compound, consequently enhancing their relative abundance. Alternatively, nonylphenol (NP) toxicity may have precipitated the elimination of specific microbial taxa or diminished their relative prevalence [43]. The elevation of α-diversity indices in N50 and M10 treatments compared to CK potentially stems from the combined interplay of multiple factors: nonylphenol (NP) serving as a supplementary carbon source, its inherent toxicity, fluctuations in soil pH, variations in soil enzyme activity, and the potent oxidative properties of MnO_2_. Meanwhile, the significant β-diversity disparities between treatment groups may be attributed to the emergence of specialized nonylphenol (NP)-degrading and Mn-oxidizing bacterial populations, ultimately precipitating shifts in bacterial species composition.

The predominant bacterial phyla identified in N50 soil throughout this investigation comprised Chloroflexi, Proteobacteria, Acidobacteriota, Firmicutes, Planctomycetota, Verrucomicrobiota, Actinobacteriota, Desulfobacterota, Gemmatimonadota, and Bacteroidota, consistent with the findings of Chen et al. [42]. These phyla constituted the dominant microbial groups within this experiment, indicating their crucial roles in nonylphenol (NP) removal and the enhancement of plant resistance. Firmicutes prevailed across all treatment cohorts, and research indicates a strong association between Firmicutes and Proteobacteria with organic matter decomposition. Similarly, Akhiladas et al. investigated refinery wastewater bioremediation in constructed wetlands, demonstrating Firmicutes and Proteobacteria as the principal phyla driving the remediation process, with genera such as Pseudomonas, Acinetobacter, and Bacillus identified as key hydrocarbon-degrading bacteria [44]. Proteobacteria exhibit a profound connection to organic matter degradation, with constituent bacteria playing pivotal roles in the breakdown and cycling of organic compounds in both natural and engineered systems. This phylum possesses exceptionally diverse metabolic capabilities, enabling the degradation of a broad spectrum of organic substrates. Takeo et al. discovered that Sphingomonas sp. Strain NP5, a Proteobacteria member, expresses a distinctive monooxygenase capable of attacking diverse 4-alkylphenols with branched side chains, thereby facilitating phenolic compound degradation [45]. Planctomycetota bacteria predominantly inhabit aquatic environments and contribute significantly to carbon and nitrogen cycling. Furthermore, Actinobacteriota genera, including Streptomyces, Rhodococcus, and Nocardia, are possibly involved in nonylphenol (NP) degradation, facilitating its breakdown into simpler intermediates through enzymatic secretion (e.g., oxygenases, and dehydrogenases), ultimately mineralizing it into carbon dioxide and water [46].

At the genus level, both N50 and M10 treatments enhanced the relative abundance of *Clostridium_sensu_stricto_12*, *Desulfitobacterium*, and *Citrifermentans* compared to the control. *Clostridium_sensu_stricto_12*, a taxon within the *Clostridium* genus of the Firmicutes phylum, encompasses bacteria capable of degrading complex organic compounds. This bacterial group predominates in the fermentation of agroforestry wastes and correlates with elevated acetic acid concentrations during fermentation [46]. *Desulfitobacterium* comprises anaerobic, sulfate-reducing proteobacteria that transform soil contaminants. These bacteria possibly involved in degrading aromatic compounds in oxygen-deprived environments, represent dominant genera, and are extensively utilized in wastewater treatment [47]. *Citrifermentans* constitutes a metabolically diverse group of anaerobic bacteria characterized by fermentative metabolism. The cultivation method of *A. donax* var. *versicolor* likely augmented the relative abundance of these anaerobic genera in the experimental setting.

The LEfSe results provide statistical support for the enrichment of specific taxa in the M10 treatment. The significant enrichment of *Desulfitobacterium* and *Clostridium_sensu_stricto_12* in M10 suggests that these anaerobic or facultative anaerobic taxa may be associated with the MnO_2_-amended rhizosphere environment. *Desulfitobacterium* is known for its ability to transform soil contaminants, and *Clostridium_sensu_stricto_12* is associated with the fermentation of complex organic compounds. However, it should be emphasized that *16S rRNA* gene sequencing only reflects relative abundance and taxonomic composition. The increased abundance of these taxa does not demonstrate their direct involvement in nonylphenol (NP) degradation. They should be regarded as treatment-associated indicator taxa, rather than functionally validated nonylphenol (NP) degraders. In future studies, functional validation can be conducted using methods such as metagenomic analysis, quantitative PCR for degradation genes, or isolation via pure culture.

### 4.3. MnO_2_ Dose–Response and Nonylphenol (NP) Removal

The reduction in extractable nonylphenol (NP) in CK1 soil was minimal and negligible. However, the cultivation of *A. donax* var. *versicolor* resulted in a substantial reduction in nonylphenol (NP) content, with final levels reaching merely 28.48% of the initial concentration. This phenomenon may be attributed to the enhanced oxidative degradation of nonylphenol (NP) in soil facilitated by MnO_2_, which intensifies redox reactions as its concentration increases [48]. MnO_2_ exerts a significant influence on soil microbial activities, organic matter decomposition, and nutrient cycling, while simultaneously stimulating the proliferation of specific soil microorganisms and the subsequent degradation of nonylphenol (NP) [49]. Furthermore, the application of an optimal quantity of MnO_2_ can defend the system of *A. donax* var. *versicolor*, bolstering its resistance to nonylphenol (NP) and thereby amplifying its capacity for nonylphenol (NP) degradation [50]. Additionally, appropriate MnO_2_ supplementation furnishes essential trace elements for plants, augmenting photosynthetic and respiratory processes, enhancing photosynthetic efficiency, facilitating organic matter accumulation, and ultimately fostering robust plant growth [51]. This enhanced plant growth stimulates the root system, causing it to exude increased quantities of secretions that serve as additional carbon sources for soil microorganisms and augment microbial diversity, consequently intensifying the microbial degradation of nonylphenol (NP) [52,53]. Concurrently, soil microbial communities exert influence over comprehensive soil properties and plant development, providing crucial ecological functions that modulate plant metabolism and hormonal pathways, directly impacting plant performance while indirectly fortifying stress tolerance, and thus facilitating the degradation of nonylphenol (NP) by *A. donax* var. *versicolor* [54,55].

### 4.4. Limitations and Implications for Integrated Remediation

This study focuses on the soil remediation effects of a combined MnO_2_–*A. donax* var. *versicolor* treatment approach. This study demonstrated that MnO_2_ supplementation increased soil respiration and urease activity, altered acid phosphatase and sucrase activities in a dose-dependent manner, and elevated the removal of extractable nonylphenol (NP) compared to the N50 group. These effects may be attributable to the combined interaction among MnO_2_, plants and their rhizosphere microorganisms. However, because an abiotic nonylphenol (NP) + MnO_2_ control without plants was not included, the relative contributions of direct MnO_2_ oxidation and microbial transformation could not be quantified. Therefore, in future studies, we can incorporate abiotic controls, time-course sampling, and functional microbial assays, and can also quantify nonylphenol (NP) in soil, roots, shoots, and transformation products to close the mass balance.

## 5. Conclusions

This investigation revealed that the introduction of 50 mg kg^−1^ nonylphenol (NP) suppressed soil respiration while concurrently elevating urease, phosphatase, and sucrase activities, resulting in detrimental effects on soil microbial communities. This research established that low MnO_2_ concentrations (≤25 mg kg^−1^) augmented soil respiration and urease activity, altered acid phosphatase and sucrase activities, and were associated with changes in soil microbial community composition. MnO_2_ supplementation minimally impacted fundamental soil physicochemical properties, but substantially altered microbial β-diversity in the M10 treatment compared with CK and N50. Within the N50 and M10 treatments, Firmicutes and Proteobacteria predominated at the phylum level, while the relative abundances of *Clostridium_sensu_stricto_12*, *Desulfitobacterium*, and *Citrifermentans* were enriched. LEfSe analysis identified *Desulfitobacterium* and *Clostridium_sensu_stricto_12* as indicator taxa for the M10 treatment, suggesting a potential association between MnO_2_ addition and the enrichment of these specific bacterial groups. This study demonstrates that the combined application of MnO_2_ and *A. donax* var. *versicolor* enhanced the removal of extractable nonylphenol (NP) from soil.

## Figures and Tables

**Table 1:** The basic physical and chemical properties of soil.

pH	Organic Carbon (g kg^−1^)	Available Nitrogen (mg kg^−1^)	Available Phosphorus (mg kg^−1^)	Available Potassium (mg kg^−1^)	Total Manganese (mg kg^−1^)
6.67	0.89	178.5	18.4	616.1	455.0

**Table 2:** Experimental design.

Treatment Groups	Soil Amount per Pot (kg)	Additives
CK	3	*A. donax* var. *versicolor* without Nonylphenol
CK1	3	50 mg kg ^−1^ Nonylphenol without *A. donax* var. *versicolor*
N50	3	*A. donax* var. *versicolor* + 50 mg kg ^−1^ Nonylphenol
M5	3	*A. donax* var. *versicolor* + 50 mg kg ^−1^ Nonylphenol + 5 mg kg ^−1^ MnO_2_
M10	3	*A. donax* var. *versicolor* + 50 mg kg^−1^ Nonylphenol + 10 mg kg^−1^ MnO_2_
M25	3	*A. donax* var. *versicolor* + 50 mg kg ^−1^ Nonylphenol + 25 mg kg ^−1^ MnO_2_
M50	3	*A. donax* var. *versicolor* + 50 mg kg ^−1^ Nonylphenol + 50 mg kg ^−1^ MnO_2_
M100	3	*A. donax* var. *versicolor* + 50 mg kg ^−1^ Nonylphenol + 100 mg kg ^−1^ MnO_2_
M250	3	*A. donax* var. *versicolor* + 50 mg kg ^−1^ Nonylphenol + 250 mg kg ^−1^ MnO_2_

**Table 3:** Effects of different treatments on α-diversity of soil bacterial communities.

Treatment Groups	OTU Number	Shannon Index	Simpson Index	Chaol Index	ACE Index
CK	2892.67 ± 61.22 a	6.81 ± 0.18 b	0.94 ± 0.01 b	3128.78 ± 61.07 a	3283.57 ± 62.5 a
N50	2879.17 ± 63.95 a	7.27 ± 0.10 a	0.95 ± 0.01 ab	3117.10 ± 64.35 a	3265.43 ± 69.60 a
M10	2995.00 ± 49.64 a	7.51 ± 0.13 a	0.96 ± 0.01 a	3217.00 ± 47.39 a	3345.15 ± 49.39 a

Note: Different lowercase letters in the table indicate significant differences between treatments in the same period (*p* < 0.05), as shown below.

## Data Availability

The original contributions presented in this study are included in the article. Further inquiries can be directed to the corresponding author.
